# Aging of the mesolimbic tract in the human brain: A diffusion tensor imaging study

**DOI:** 10.1097/MD.0000000000030924

**Published:** 2022-10-14

**Authors:** Jeong Pyo Seo, Heun Jae Ryu

**Affiliations:** a Department of Physical Therapy, College of Health and Welfare Sciences, Dankook University, Cheonan, Republic of Korea; b Department of Public Health Sciences, Graduate School, Dankook University, Cheonan, Republic of Korea.

**Keywords:** aging, diffusion tensor tractography, dopaminergic pathway, mesolimbic tract, social interaction

## Abstract

The mesolibic tract (MLT) is a dopaminergic tract that has been shown to play a role in regulating reward stimuli, including both incentive salience and social stimuli. In the current study, we examined the aging of MLT in normal human participants to explain human brain structures using diffusion tensor tractography (DTT). Fifty-seven healthy participants were recruited for this study and allocated to six groups based on their age. Diffusion tensor imaging (DTI) scanning was performed and MLTs were reconstructed using the probabilistic tractography method. MLTs were defined by selecting fibers passing through the seed and target regions of interest placed on the ventral segmental area and nucleus accumbens. A significant negative correlation was observed between age and the voxel number (VN) of MLT, while a positive correlation was observed between age and the apparent diffusion coefficient (ADC). The mean VN value of the MLT was significantly lower in the 60s and 70s age groups than in the 20s, 40s, and 50s (*P* < .05). The mean ADC value of the MLT was significantly higher in the 60s and 70s groups than in the 20s, 30s, and 40s, 50s groups (*P* < .05). We found that aging of the MLT began in the 20s or 30s and progressed steadily throughout life until the 60s, when it exhibited significant degeneration. We believe this affect may play a role in the decline of memory and social interaction with aging in normal participants.

## 1. Introduction

Dopamine is released to the nucleus accumbens (NAc) from the ventral tegmental area (VTA), and has been shown to play a role in regulating reward stimuli, including not only incentive salience related to addiction, reinforcement learning, and motivation, but also social stimuli, such as social anticipated reward over monetary and social interaction.^[1–4]^ The NAc plays a role in controlling motivated behaviors, such as eating and drinking, which includes a desire or wanting. On the other hand, social stimuli like anticipated monetary reward made NAc only activate fMRI, as compared with age, sex, and methods.^[5]^

The mesolimbic tract (MLT) connects from the VTA of the midbrain to the NAc of the ventral striatum, and is one of the reciprocal dopaminergic pathways that links to the medial prefrontal bundle.^[6,7]^ The VTA in the midbrain includes dopaminergic neurons projecting chiefly to the NAc.^[7]^ Impairment or injury of the MLT in the human brain causes impaired control over behavior, craving, withdrawal, and tolerance, which leads to non-drug addiction such as sexual, gambling, shopping, and video game addiction. The impairment of the MLT, comparing normal children with children with autism, also causes social interaction impairments.^[3]^ Moreover, the MLT is a part of dopaminergic circuits and the circuitry, as aging has been known to cause a decline in a number of cognitive processes, including working and episodic memory and attention. Therefore, these processes show a strong correlation with MLT.^[8–14]^

As detailed knowledge on the participant of normal aging of precise brain structures assists in the development of strategies with the intent of curbing or deferring aging, diffusion tensor tractography (DTT) can be used to perform three-dimensional qualitative visualization and analysis of neural structures so that these contemporary technique studies have been performed.^[15–20]^ However, few such studies have been conducted to resolve the effect of aging on MLT. In the current study, we investigated the aging effect of MLT in the normal human brain using DTT.

## 2. Methods

### 2.1. Participants

Fifty-seven right-handed, healthy participants (men: 33, women: 24, mean age: 47.11 ± 17.24 years; range: 20–78 years) with no previous history of neurological, psychiatric, or physical illness and no brain lesions on conventional MRI (T1-weighted, T2-weighted, Fluid attenuated inversion recovery [FLAIR] or T2-weighted gradient recall echo [GRE] images), as confirmed by a neuroradiologist, were enrolled in the present study. The participants were divided into six groups by age. All participants provided written informed consent prior to study commencement, and the study protocol was approved by the institutional review board of the university hospital.

### 2.2. Diffusion tensor imaging (DTI) acquisition

DTI data were acquired using a Synergy-L SENSE head coil on a 1.5T Gyroscan Intera system (Philips, Best, The Netherlands), equipped with single-shot echo-planar imaging. For each of the 32 non-collinear diffusion sensitizing gradients, 67 contiguous slices were acquired parallel to the anterior commissure-posterior commissure line. The imaging parameters were as follows: acquisition matrix = 96 × 96, reconstructed matrix = 192 × 192 matrix, field of view = 240 × 240 mm^2^, TR = 10,398 ms, TE = 72 ms, parallel imaging reduction factor (SENSE factor) = 2, EPI factor = 59 and b = 1000 s/mm^2^, NEX = 1, slice gap = 0, and slice thickness 2.5 mm.

### 2.3. Fiber tracking

The Oxford Centre for Functional Magnetic Resonance Imaging of the Brain (FMRIB) software library was used to analyze the diffusion-weighted imaging data.^[21,22]^ The DTT software CMRM (Jogns Hopkins Medical Institute, Baltimore, MD) was used to evaluate the neural tracts. Fiber tracking was based on the fiber assignment continuous tracking (FACT) algorithm and multiple regions of interest (ROIs) approach.

**Figure 1.**
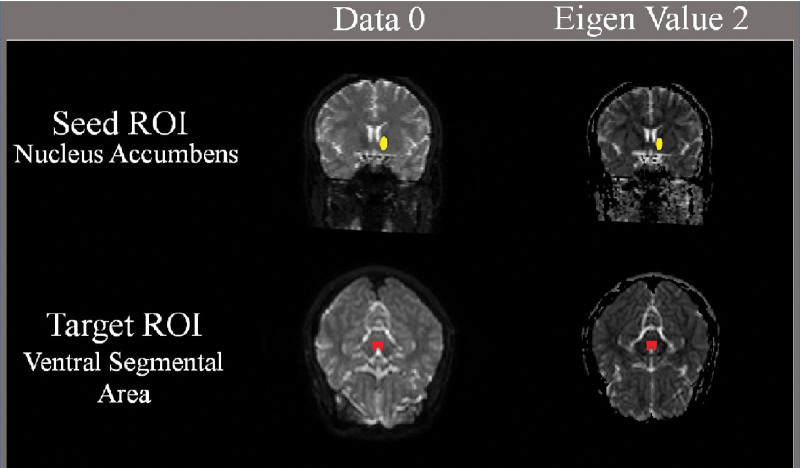
Seed regions of interest (ROIs) for mesolimbic tract were placed on the ventral tegmental area (red). Target ROIs for mesolimbic tract were placed on the nucleus accumbens (yellow).

The MLT was delineated by selecting fibers that passed through the seed and target regions of interest (ROI). For each participant, the first ROI was located at the NAc of the ventral striatum on the data 0 map, and the second ROI was placed on the ventral tegmental area on the eigen value 2 map in the midbrain (Fig. 1). The fiber tracking started at the center of a seed voxel with a fractional anisotropy (FA) of <0.15 and a tract turning angle of <70°. DTI parameters, including the voxel number (VN), apparent diffusion coefficient (ADC), and FA of the MLT were measured.

### 2.4. Statistical analysis

Data were analyzed using SPSS software (version 25.0; SPSS Inc., Chicago, IL). Pearson’s correlation analysis was performed to assess the significance of correlations between these three DTI parameters (VN, ADC, and FA) and age. Multiple ANOVA with the LSD post hoc test was used to determine the significance of differences for each DTI parameter (VN, ADC, and FA) between age groups. Statistical significance was set as *p* values < .05.

## 3. Results

**Figure 2.**
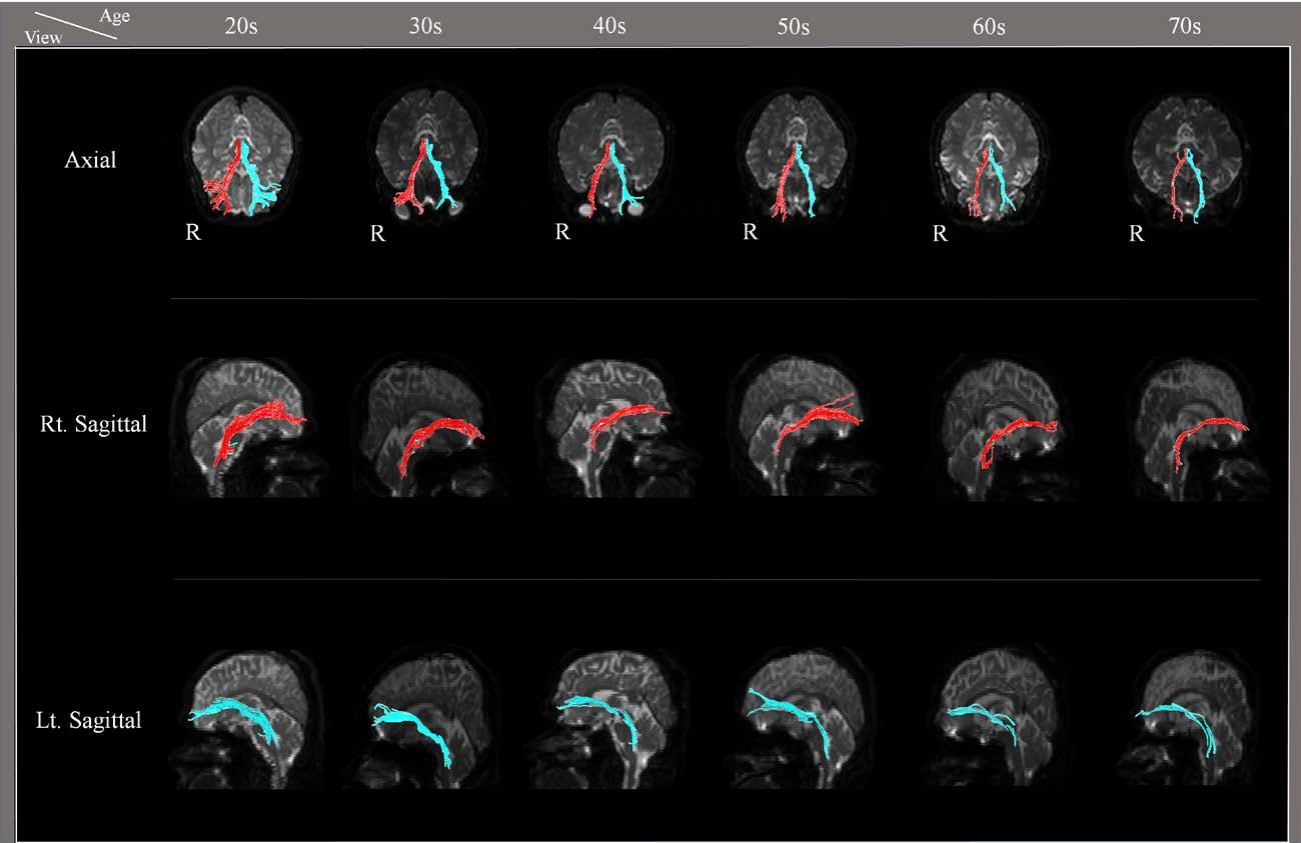
The reconstructed mesolimbic tracts derived depending to age.

Correlations between age and DTI parameters were as follows. We observed a moderate negative correlation between the age and VN of the MLT (r = −0.743, *P* < .05) and a weak positive correlation between age and ADC values of the MLT (*R* = 0.392, *P* < .05). In contrast, age was not correlated with MLT FA (r = −0.166, *P* > .05).^[23]^ Results of multiple-ANOVA showed significant differences in both the VN and ADC of the MLT between age groups (*P* < .05) (Fig. 2) (Table 1). The mean VN value of the MLT was higher in the 20s and 30s age groups than in the other groups, as well as in the 40s and 50s groups than in the 60s and 70s groups (*P* < .05). The mean ADC value of MLT was lower in the 20s and 30s groups than in the 60s and 70s groups, in the 40s and 50s groups than in the 70s group, and greater in the 60s group than in the 20s and 30s groups, and in the 70s group than in the 60s group (*P* < .05). However, the mean FA values of the MLT showed no significant differences between the age groups (*P* > .05).

**Table 1:** Mean values of diffusion tensor image parameters of the mesolimbic tract.

		20s	30s	40s	50s	60s	70s	*p* value
MLT	VN	1274.71	1180.38	827.56	677.64	474.30	455.63	0.000*
		(±399.00)	(±335.57)	(±213.58)	(±214.28)	(±144.14)	(±164.29)	
	ADC	0.7807	0.7838	0.8008	0.790314	0.815680	0.8374	0.001*
		(±0.03)	(±0.03)	(±0.04)	(±0.04)	(±0.05)	(±0.07)	
	FA	0.4480	0.4320	0.4357	0.4428	0.4307	0.4323	0.150
		(±0.02)	(±0.02)	(±0.03)	(±0.02)	(±0.04)	(±0.02)	

ADC = apparent diffusion coefficient, FA = fractional anisotropy, MLT = mesolimbic tract, VN = voxel number.

Values indicate mean (±standard deviation).

* *P* < .05 MANOVA.

## 4. Discussion

In this study, we investigated the aging of MLT in the normal human brain by diffusion tensor imaging. In accordance with our findings, we concluded that: our results show an overall decreasing trend in the VN, and an increasing trend in the ADC of the MLT with aging; this signifies a gradient of decline in the VN values of the MLT of the 60s and 70s groups compared with those in the 20s, 30s, 40s, and 50s age groups; and the VN of the MLT was decreased in the 40s age groups compared with that of the 20s and 30s age groups, but not in the 50s age group. An increase was observed in the ADC values of the MLT of the 60s and 70s groups compared with those of the 20s and 30s age groups.

The VN values indicate the total number of fibers in the neural tract.^[24]^ The ADC values reflect the magnitude of water diffusion. In contrast, the FA value indicates the degree of directionality and integrity of white matter microstructures, such as axons, myelin, and microtubules.^[25,26]^ Age-related declines in VN values and inclines in ADC values may indicate demyelination, and reductions in the number of myelinated fibers in a neural tract could reduce the VN values.^[19,25,27]^ Therefore, our study shows that the degenerative process of aging begins in the 20s or 30s, progresses steadily at a nearly continuous rate over the lifespan, with a significant degenerative aging effect in the MLT occurring in the 60s.

According to some previous studies, age-related changes in the MLT were affected by dopaminergic manipulation; hence, dopaminergic circuits with aging have a strong correlation with the decline of memory.^[8,9,13,14,28]^ In addition, MLT has been highlighted as having an important relationship with social play behavior, such as reward-associated processes, cognitive control, decision making, and behavioral inhibition.^[29,30]^ On the other hand, other results showed that normal elders exhibit a reduction in their social activity and interest, which may be caused by a decline in social stimuli reward relevant to the aging of the MLT.^[31]^ Consequently, our findings might be able to extend these previous studies by showing that the connectivity of the structural tract between the mesolimbic pathway is not only debilitated in older adults, but is also related to aging differences in encoding memory and social interaction.

In conclusion, using DTT, we identified that the MLT links to the NAc from the VTA in the normal human brain, and further found that aging of the MLT begins in the 20s or 30s and progresses steadily at a nearly continuous rate throughout life, until the 60s, when degeneration of the MLT increases significantly compared to the rate of those in their 20s or 30s. We believe that our findings may, at least partially, explain the decline in memory and social interaction with aging. To the best of our knowledge, studies to date have not systematically examined the formational connectivity of the MLT connecting the VTA to the NAc in the human brain, which is related to aging. Therefore, this is the first study to demonstrate the aging effect of MLT using DTT, and we believe that this finding will aid clinicians in the field of human aging and neuroscience. However, some limitations of this study should be considered. First, there is a lack of data on individuals older than 80 years and clinical data related to MLT function. Second, we could not recruit any individuals older than 80 years of age. Therefore, further prospective studies should be conducted involving individuals older than 80 years and neurobehavioral data should be collected to enable adequate assessment of MLT function.

**Figure 3.**
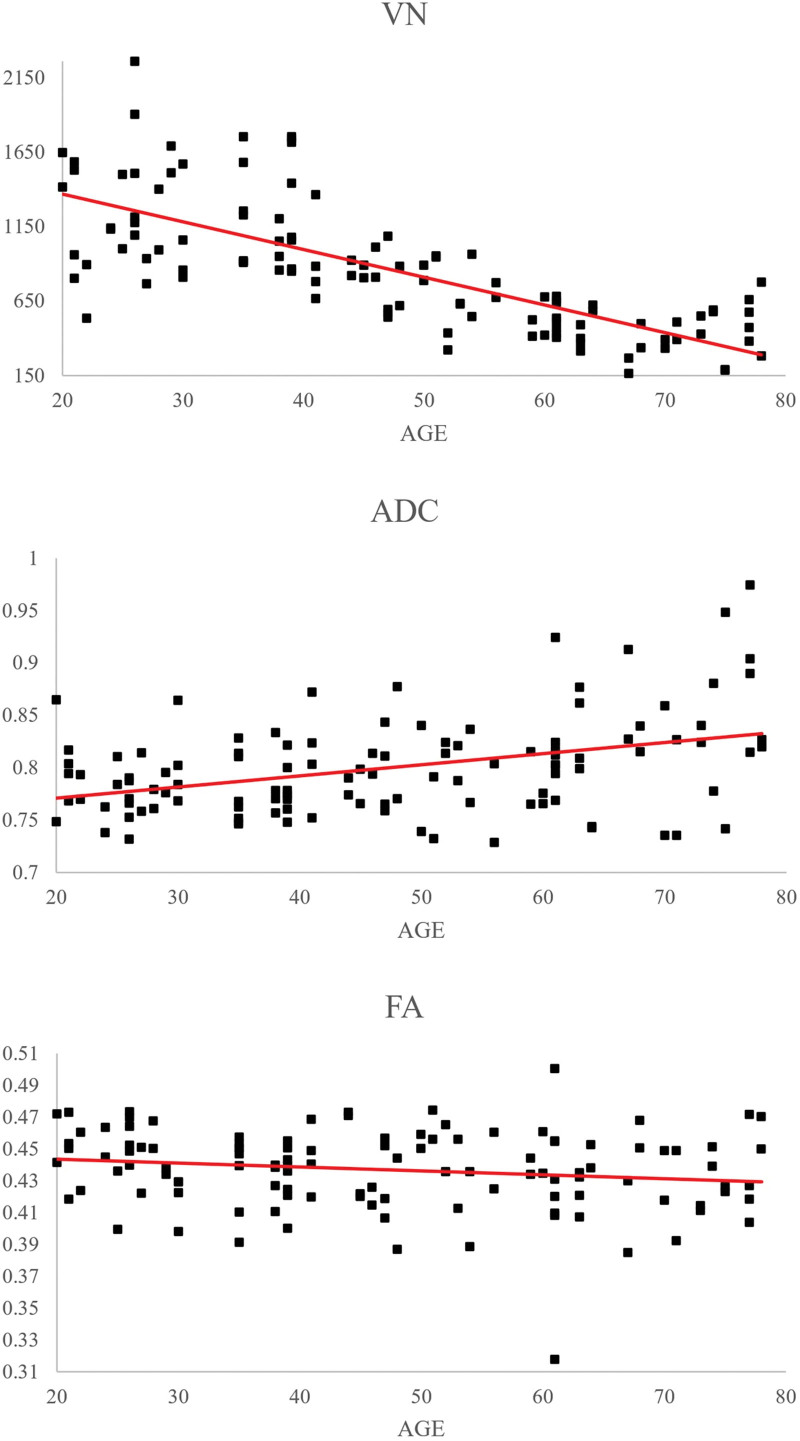
Correlations between age and the three DTT parameters. DTT = diffusion tensor tractography.

## Acknowledgments

This work was supported by the National Research Foundation (NRF) of Korea Grant funded by the Korean Government (MSIP) (2021R1F1A1064353).

## Author contributions

Conceptualization: Jeong Pyo Seo.

Data curation: Jeong Pyo Seo.

Investigation: Heun Jae Ryu.

Methodology: Heun Jae Ryu.

Validation: Jeong Pyo Seo.

Visualization: Heun Jae Ryu.

Writing – original draft: Heun Jae Ryu.

Writing – review & editing: Jeong Pyo Seo.
